# Protocol for AREST: Apixaban for Early Prevention of Recurrent Embolic Stroke and Hemorrhagic Transformation—A Randomized Controlled Trial of Early Anticoagulation After Acute Ischemic Stroke in Atrial Fibrillation

**DOI:** 10.3389/fneur.2019.00975

**Published:** 2019-09-20

**Authors:** David Z. Rose, John N. Meriwether, Michael G. Fradley, Swetha Renati, Ryan C. Martin, Thomas Kasprowicz, Aarti Patel, Maxim Mokin, Ryan Murtagh, Kevin Kip, Andrea C. Bozeman, Tara McTigue, Nicholas Hilker, Bonnie Kirby, Natasha Wick, Nhi Tran, W. Scott Burgin, Arthur J. Labovitz

**Affiliations:** ^1^Department of Neurology, Morsani College of Medicine, University of South Florida, Tampa, FL, United States; ^2^Department of Cardiology, Morsani College of Medicine, University of South Florida, Tampa, FL, United States; ^3^Department of Radiology, Morsani College of Medicine, University of South Florida, Tampa, FL, United States; ^4^College of Public Health, University of South Florida, Tampa, FL, United States

**Keywords:** acute ischemic stroke, atrial fibrillation, anticoagulation timing, direct oral anticoagulant, apixaban, transient ischemic attack

## Abstract

**Background:** Optimal timing to initiate anticoagulation after acute ischemic stroke (AIS) from atrial fibrillation (AF) is currently unknown. Compared to other stroke etiologies, AF typically provokes larger infarct volumes and greater concern of hemorrhagic transformation, so seminal randomized trials waited weeks to months to begin anticoagulation after initial stroke. Subsequent data are limited and non-randomized. Guidelines suggest anticoagulation initiation windows between 3 and 14 days post-stroke, with Class IIa recommendations, and level of evidence B in the USA and C in Europe.

**Aims:** This open-label, parallel-group, multi-center, randomized controlled trial AREST (Apixaban for Early Prevention of Recurrent Embolic Stroke and Hemorrhagic Transformation) is designed to evaluate the safety and efficacy of early anticoagulation, based on stroke size, secondary prevention of ischemic stroke, and risks of subsequent hemorrhagic transformation.

**Methods:** Subjects are randomly assigned in a 1:1 ratio to receive early apixaban at day 0–3 for transient ischemic attack (TIA), 3–5 for small-sized AIS (<1.5 cm), and 7–9 for medium-sized AIS (1.5 cm or greater but less than a full cortical territory), or warfarin at 1 week post-TIA or 2 weeks post-stroke. Large AISs are excluded.

**Study Outcomes:** Primary: recurrent ischemic stroke, TIA, and fatal stroke; secondary: intracranial hemorrhage (ICH); hemorrhagic transformation (HT) of ischemic stroke; cerebral microbleeds (CMBs); neurologic disability [e.g., modified Rankin Scores (mRS), National Institutes of Health Stroke Scale (NIHSS), Stroke Specific Quality of Life scale (SS-QOL)]; and cardiac biomarkers [e.g., AF burden, transthoracic echo (TTE)/transesophageal echo (TEE) abnormalities].

**Sample Size Estimates:** Enrollment goal was 120 for 80% power (two-sided type I error rate of 0.05) to detect an absolute risk reduction of 16.5% postulated to occur with apixaban in the primary composite outcome of fatal stroke/recurrent ischemic stroke/TIA within 180 days. Enrollment was suspended at 91 subjects in 2019 after a focused guideline update recommended direct oral anticoagulants (DOACs) over warfarin in AF, excepting valvular disease (Class I, level of evidence A).

**Discussion:** AREST will offer randomized controlled trial data about timeliness and safety of anticoagulation in AIS patients with AF.

**Clinical Trial Registration:** www.ClinicalTrials.gov, identifier NCT02283294.

## Introduction, Background, and Aims

For patients with acute ischemic stroke (AIS) due to atrial fibrillation (AF), it is currently unknown when the optimal time is to prescribe oral anticoagulation. Early use may prevent secondary ischemic stroke from AF but may provoke hemorrhagic transformation (HT). Later use may prevent HT, but unanticoagulated AF can provoke further AIS, particularly in patients at higher risk based on CHADSVASc score. Early initiation of oral anticoagulation may mitigate the exorbitant fivefold recurrent risk of AIS seen in AF (when compared to other stroke etiologies)—a risk that is especially highest within the first few weeks post-stroke (1–3). Although early anticoagulation may result in fewer ischemic events, the much-feared potential harm (i.e., bleeding) counterbalancing this benefit has never been formally evaluated in a randomized, clinically controlled manner and may be overestimated.

Typically, AF also results in greater infarct volumes, again compared to other etiologies of stroke. Hence, use of early anticoagulation upon an unstable, fragile, neuronal infarct bed poses a potentially greater risk of HT than waiting a few days (or weeks) for it to mature. Unsurprisingly, HT itself results in increased morbidity and mortality via mass effect, cerebral edema, and herniation syndromes, which can be fatal (1, 2).

Treatment guidelines have emerged without much firm evidence on this topic and only offer vague suggestions for timing of anticoagulation initiation in AF-related AIS. The American College of Chest Physicians (2012) and the American Heart/Stroke Association (2014) propose initiation within 2 weeks of a cardioembolic stroke, except in cases of large strokes, uncontrolled hypertension, or bleeding conditions (4, 5). The European Society of Cardiology guidelines (2016) are slightly less nebulous, with a marginally more specific window of 3–12 days, except for a list of highest-risk individuals similar to the other associations' suggestions (6). Consensus, rather than any randomized prospective data, produced these Class IIa recommendations, with level of evidence B in the USA and C in Europe.

Historically, opinion about oral anticoagulation timing for AF-related AIS has been based on experience with warfarin. With the interval development of better-tolerated direct oral anticoagulant (DOAC) therapy, which takes only a few hours to reach therapeutic level in the blood, not days like warfarin does, the optimal timing of anticoagulation initiation after an ischemic stroke is even more unclear—yet even more necessary given the rapid adoption of the DOAC class for primary and secondary stroke prevention from AF. Non-randomized and openly confounded studies have inferred safety and tolerability of early DOAC use post-stroke from AF (7–11); however uncertainty exists about the actual risk–benefit ratio and the appropriate strategic latency required before anticoagulation initiation in these fragile patients.

Given the clinical equipoise in this at-risk population, and to address this grossly unmet need, the neurology and cardiology departments at the University of South Florida (vis-à-vis our “USF Neuro-Cardiac Program”) designed the randomized, controlled AREST trial: Apixaban for Early Prevention of Recurrent Embolic Stroke and Hemorrhagic Transformation. We hypothesize that in patients with non-valvular AF and AIS, initiating early oral anticoagulation with the DOAC apixaban (precise timing based on actual stroke size) results in fewer recurrent ischemic strokes or transient ischemic attacks (TIAs), without significantly increasing the risk of intracranial hemorrhage (ICH), vs. warfarin initiated at 14 days (9–11).

## Methods

### Design

**Figure 1:**
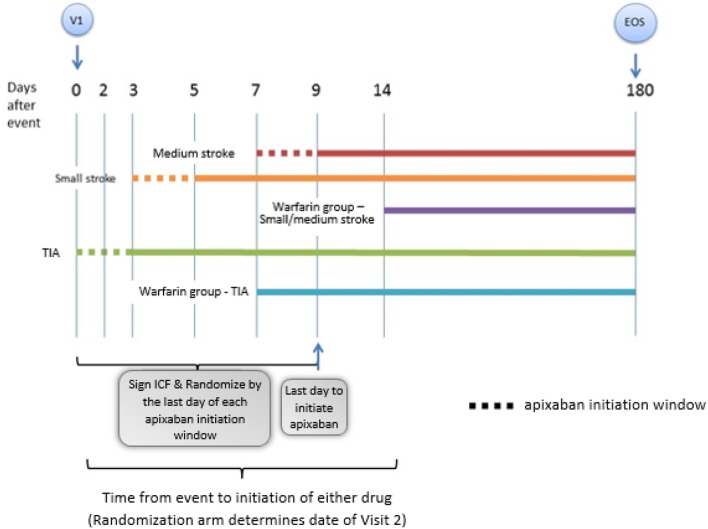
This is a representation of the general study schema for the AREST (Apixaban for Early Prevention of Recurrent Embolic Stroke and Hemorrhagic Transformation) study (V1 = visit 1, time 0; EOS = end of study, day 180). Details of the follow up visits entailed in the follow up section of the proof of concept.

AREST is an open-label, parallel-group, randomized, active controlled trial designed to examine the effect of early initiation of the DOAC apixaban in AF at day 0–3 for TIA, 3–5 for small-sized AIS, and 7–9 for medium-sized AIS, vs. warfarin at 1 week post-TIA or 2 weeks post-stroke. Figure 1 shows the study flowchart. Subjects are prospectively identified, give consent, and are enrolled by the Neuro-Cardiac Program team at the University of South Florida in Tampa, with additional sites including Bayfront Health in St. Petersburg, University of Louisville in Kentucky, and University of California-Los Angeles. The diagnosis of AIS, ICH, HT, and cerebral microbleed (CMB) was adjudicated by committee; imaging discrepancies were resolved by investigator consensus.

### Patient Population, Inclusion, and Exclusion Criteria

Eligible patients are those ≥18 years of age who have experienced AIS within 3–5 days, or TIA within 3 days, and have AF by history, electrocardiogram (EKG), telemetry, or longer-term cardiac monitor. Electrographic criteria include irregular atrial activity without distinct regular recurring P-wave and an irregularly irregular R-to-R interval if atrial-to-ventricular conduction is intact. Mode switch episodes recorded by implantable pacemakers and defibrillators were insufficient to diagnose AF, if unaccompanied by corresponding intracardiac electrograms. An electrophysiologist reviewed recorded arrhythmias and confirmed AF. Although neuroimaging in some potential subjects may have suggested cardioemboli, AREST enrolled neither cryptogenic stroke nor embolic stroke of undetermined source (ESUS). Generally, without cardiac monitoring, there may be an overdiagnosis of cryptogenic strokes and underdiagnosis of AF (12); however, we permitted only a clear and convincing past medical history of AF or a newly identified AF rhythm pattern for study entry.

Strokes were defined by the presence of focal neurological deficits and classified by neuroradiographic size of the largest area of AIS. Neuroimaging utilized diffusion-weighted image (DWI) sequences of brain magnetic resonance imaging (MRI), or head computed tomography (CT) if the patient was MRI-incompatible (pacemaker, claustrophobia, etc.). By definition, TIA must have resolved focal deficits without acute DWI changes on MRI or hypodensities on CT. The clinical classification of AIS was based on the Oxfordshire Community Stroke Project (OCSP) for predicting the site and size of AIS on imaging and has previously been validated (13). Small-sized AIS can involve the anterior or posterior circulation and is <1.5-cm in largest dimension. Medium-sized AIS is ≥1.5 cm but less than a full cortical territory of the anterior cerebral artery (ACA), middle cerebral artery (MCA), or posterior cerebral artery (PCA). Large-sized AIS (complete arterial territory) and brainstem strokes are excluded due to potentially excessive (and fatal) HT risk from early anticoagulation.

Other exclusion criteria were primary ICH, HT of the AIS, or AIS from other etiologies (i.e., ipsilateral carotid stenosis). Subjects could be enrolled with ≤ 3 CMBs on gradient recovery echo (GRE) sequence on MRI—or susceptibility weighted imaging (SWI) if GRE is unavailable. Subjects were excluded if MRI detected CMBs within or adjacent to the infarct, or >3 CMBs anywhere in the brain (cortical or subcortical); these restrictions were created to avoid the potentially higher risk of CMB expansion when starting anticoagulation.

Further exclusion criteria include therapeutic anticoagulation at the time of admission [active warfarin use with admission International Normalized Ratio (INR) ≥2.0 or administered two consecutive doses of DOAC] or another need for anticoagulation [e.g., mechanical valve, Deep Vein Thrombosis (DVT), hypercoagulable state] or dual anti-platelet therapy (e.g., cardiac stent); major bleeding within the last 6 months; blood dyscrasias; traumatic brain injury (TBI)–associated ICH within 1 year; blood pressure ≥180/100 mm Hg on day of randomization [per Principal Investigator (PI) discretion]; illicit drug and/or alcohol use (per PI discretion); liver disease (defined as liver enzymes twice the upper limit); end stage renal disease; concurrent use of specific dual inhibitors of CYP3A4 and P-glycoprotein; anemia with hemoglobin <9 gm/dl; thrombocytopenia with platelet count <100 K/μl; or pregnant/lactating women (must have a negative pregnancy test prior to enrollment).

### Randomization and Intervention

Subjects are randomly assigned in a 1:1 ratio to either apixaban or warfarin. Prior to discharge, subjects receive neuroimaging, arterial studies of the head and neck, and transthoracic echo (TTE) with or without transesophageal echo (TEE). A 30-day cardiac event monitor may be given at discharge or afterwards. Subjects are followed at the following intervals: post-stroke/TIA days 14, 30, 60, 90, 120, 150, and 180. An end-of-study phone interview occurs at day 210. At follow-up visits, subjects receive vitals, intermittent history, EKG, event monitor review, physical examination, NIH Stroke Scale (NIHSS), modified Rankin Scores (mRS), compliance assessment (medication review), and adverse event assessment. At baseline, days 30 and 180 (or early withdrawal) visits, a Stroke Specific Quality of Life scale (SS-QOL) is collected. At baseline and days 30, 90, and 180, labs are collected (lipid panel, coagulation studies, comprehensive metabolic panel, and complete blood count). MRI (or CT if MRI-incompatible) is performed at baseline and days 14 and 180 (or early withdrawal).

### Study Outcomes

Primary end points include a composite of recurrent ischemic stroke, TIA, and fatal stroke at days 30 and 180 (primary efficacy outcome). Secondary end points are ICH (primary safety end point), which can include primary hemorrhagic stroke, HT of ischemic stroke, or new CMB on GRE or SWI. Follow-up continues until day 180 from the index stroke event or until one of the primary or secondary end points is reached—at which time the subject completes the study. Additional outcome data include mRS and NIHSS for Neurologic Disability, SS-QOL, TTE/TEE abnormalities, and AF burden on cardiac event monitoring.

### Sample Size Estimates

The enrollment goal was 120 patients in order to provide 80% power (two-sided type I error rate of 0.05) to detect a large absolute risk reduction of 16.5% postulated to occur with apixaban in the primary composite outcome of fatal stroke/recurrent ischemic stroke/TIA at 180 days. Enrollment was suspended at 91 subjects in early 2019 after a focused guideline update recommended DOACs over warfarin in AF, excepting valvular disease (Class I, level of evidence A).

### Statistical Analyses

For the primary composite end point, the proportion of patients experiencing the outcome will be computed along with the absolute risk difference by random assignment and corresponding 95% confidence interval. Similarly, the number needed to treat (NNT) and 95% confidence interval will be calculated based on the incidence rates of the primary composite outcome in the two treatment groups. Because random assignment is stratified by stroke severity (small stroke, medium stroke, TIA), balanced distribution is expected. Given the modest sample size, potential unexpected confounding of baseline covariates (imbalanced distributions by random assignment) will be assessed by the use of Student *t*-tests or Wilcoxon tests for continuous variables and chi-square tests for categorical variables, conservatively using a *p*-value of <0.15. A general log-linear model will be fit with clinical site as a random effect, random assignment as the primary predictor (main effect) of interest, adjustment for any potential confounders, and the primary composite end point as the outcome variable. This will result in an adjusted risk ratio and 95% confidence interval for apixaban vs. warfarin. Separate models will be fit at 30 and 180 days. The intent-to-treat principle will be used in all analyses. For assessment of safety, the incidence of ICH will be compared by random assignment.

### Compliance With Ethical Standards

AREST has been approved by the Institutional Review Board at the University of South Florida Morsani College of Medicine and at Tampa General Hospital as well as all participating study hospitals. All study procedures are in accordance with the provisions of the International Conference of Harmonization Good Clinical Practice and the 1964 Declaration of Helsinki and its later amendments. Written informed consent is obtained from each patient (or proxy) before enrollment. AREST is registered on ClinicalTrials.gov (NCT02283294).

### Safety and Data Monitoring Body

No safety issues are expected. Any serious study-related events, along with the events captured as secondary study outcomes, will be reported to the independent Data Safety Monitoring Board (DSMB) committee.

### Study Organization and Funding

AREST is an initiative of the University of South Florida Morsani College of Medicine, in conjunction with the University of Louisville in Kentucky; Bayfront Health in St. Petersburg, Florida; and the University of California-Los Angeles. This randomized study is funded by the Bristol Myers Squibb/Pfizer Alliance.

## Collaboration

AREST was the brainchild of the academic vascular neurology and cardiology services at the University of South Florida (USF) Morsani College of Medicine. In collaboration with our 1,018-bed teaching facility, Tampa General Hospital, USF established a Neuro-Cardio Program (NCP) to create trials such as AREST and to address mutual patients' unmet needs. Starting NCP was a long sought-after effort with three major goals: (1) co-identification and co-enrollment of subjects into clinical trials that may benefit both specialties; (2) co-consultation on patients with overlapping diagnoses in a Thursday clinic with “freeze-and-thaw” time slots for mixed use; and (3) Tuesday radiology conferences for assessment of brain MRIs for CMB, HT, or other neuro-abnormalities, and echocardiograms for thrombi, valvular disease, or other cardio-abnormalities. We have found that interdisciplinary interaction at USF has led to optimal decisions on cases of neurocardiogenic syncope, insertion of implantable loop recorders or telemetry patches looking for AF in cryptogenic stroke, closure of patent foramen ovale (PFO) for paradoxical embolic ischemic stroke, AF treatment in patients unsuitable for long-term anticoagulation and shared decision-making (SDM) for left atrial appendage closure device implantation. At our monthly NCP logistical/planning meetings, we review treatment algorithms and protocols for our mutual patient population, assess progress of our clinical trials and database collection, and fix any inpatient/outpatient co-management obstacles. Without NCP, local investigator-initiated trials such as AREST are more difficult to foster, design, fund, and complete.

## Discussion

The late, great, centenarian neurologist Dr. C. Miller Fisher once quipped, “If you are treating even one patient in your practice with anticoagulation, you should wake up once a week or so in a cold sweat” (14).

For decades, the infamous vitamin-K antagonist (VKA) warfarin has been the cornerstone of stroke prevention in AF; however, the panoply of drawbacks of VKA make it cumbersome to take and manage (15, 16). Regardless, VKA is still abundantly prescribed for AF in the United States and around the world, mostly due to its inexpensive direct cost. DOACs are priced higher but may result in a significantly shorter length of stay and decreased overall and indirect medical costs compared to warfarin (17). Moreover, DOACs have shown non-inferiority (or in some cases superiority) to VKA in ischemic stroke prevention for AF as well as significantly less bleeding, especially in the intracranial bed (15, 16, 18, 19). The initial DOAC-vs.-warfarin randomized controlled trials for stroke prevention in AF, published between 2009 and 2013, did not test early use of either the study drug or its comparator, so any advantage in ischemic stroke risk reduction (or bleeding risk) during this initial time period remains unknown (7, 18–20).

These seminal clinical trials delayed anticoagulation weeks to months post-stroke, presumably due to a perceived higher HT risk vs. any potential benefit of very early secondary stroke prevention from either VKA or DOAC (15, 16, 18, 19). Undoubtedly, however, treatment initiation delay has consequence: about 10% of patients with AF-related stroke do experience recurrent embolic events within the first 30 days (7, 8, 19, 20), with immediate (<2 weeks) risk ranging from 0.1 to 1.3% per day (9). This “threshold delay,” described in 2015 by the VISTA (Virtual International Stroke Trials Archive) collaborators, represents the time point after AF–stroke when the value of anticoagulation switches from neutral (or even harmful) to beneficial (8). Prospective observational and non-randomized cohort data suggest that early initiation of anticoagulation may reduce embolic risk between 4 and 14 days post-stroke (9) and that anticoagulation started 2–3 days post-stroke, generally, could be ideal (8). In 2016, a small, prospective, open-label, MRI study of 60 patients with AF treated early (median of 3 days) with the DOAC rivaroxaban after TIA or small- to medium-sized stroke (median NIHSS of 2), identified three new HT and five HT progressions, all reportedly asymptomatic (7). Larger ischemic strokes, however, pose greater fear for HT with anticoagulation, as multivariate analysis revealed an association of higher rates of symptomatic ICH as well as AIS recurrence, interestingly (9).

Current clinical practice is variable for starting anticoagulation in AIS with AF. Guidelines offer only Class IIa recommendations, wide-open windows between 3 and 14 days, and level of evidence B in the USA and C in Europe (4–6). The risk of HT (and ICH) on an anticoagulant may be highest early, although it remains unknown and must be balanced against the benefit of reduction in recurrent ischemic strokes (16, 18). A focused guideline update in 2019 for stroke prevention in AF recommended DOACs over warfarin, excepting valvular disease (Class I, level of evidence A) (21), so AREST enrollment was suspended soon after this pronouncement. Regardless, as a randomized, controlled, multi-center trial, AREST will soon proffer some practical evidence concerning the safety and efficacy for early use of anticoagulation after AIS in AF patients while simultaneously balancing the inherent danger of AIS converting into HT as well as the risk of CMB development and progression into hemorrhagic strokes.

## Ethics Statement

The studies involving human participants were reviewed and approved by University of South Florida Institutional Review Board/Institutional Ethics Committee. The patients/participants provided their written informed consent to participate in this study.

## Author Contributions

DR, JM, RM, BK, WB, and AL contributed to study conception, design, writing, and reviewing the manuscript. MF, SR, TK, AP, MM, RCM, AB, TM, NH, NW, and NT contributed to manuscript review. KK contributed to statistical aspects of the protocol.

### Conflict of Interest Statement

DR receives modest honoraria for consulting or speaker bureau from Boehringer-Ingelheim and Medtronic as well as significant honoraria from Boston Scientific. MF receives modest honoraria for consulting from Novartis Pharmaceuticals. The remaining authors declare that the research was conducted in the absence of any commercial or financial relationships that could be construed as a potential conflict of interest.
